# Clinical Outcomes after Multivalve Surgery in Octogenarians: Evaluating the Need for a Paradigm Shift

**DOI:** 10.3390/jcm13030745

**Published:** 2024-01-27

**Authors:** Ali Taghizadeh-Waghefi, Asen Petrov, Sebastian Arzt, Konstantin Alexiou, Sems-Malte Tugtekin, Klaus Matschke, Utz Kappert, Manuel Wilbring

**Affiliations:** 1 Medical Faculty “Carl Gustav Carus”, Technical University of Dresden, 01307 Dresden, Germanymanuel.wilbring@herzzentrum-dresden.com (M.W.); 2 Center of Minimally Invasive Cardiac Surgery, University Heart Center Dresden, Medical Faculty of the Technical University of Dresden, 01037 Dresden, Germany

**Keywords:** multivalve disease, multivalve surgery, octogenarians

## Abstract

(1) Background: this study addresses the lack of comprehensive research on outcomes in octogenarians undergoing cardiac surgery for multivalvular disease, emphasizing the need for a critical examination of the intervention’s overall worth in this aging population. (2) Methods: By analyzing short-term and mid-term data from 101 consecutive octogenarian patients undergoing multivalve surgery, the study identifies predictors for in-hospital and one-year mortality. (3) Results: In-hospital mortality increased fourfold with the occurrence of at least one postoperative complication. Octogenarians undergoing multivalve surgery experienced an in-hospital mortality rate of 13.9% and an overall one-year mortality rate of 43.8%. Postoperative delirium was identified as an independent risk factor, contributing to elevated risks of both in-hospital and one-year mortality. Prolonged surgical procedure time emerged as an independent risk factor associated with increased in-hospital mortality. Continuous veno-venous hemodialysis showed an independent impact on in-hospital mortality. Both re-intubation and the transfusion of packed red blood cells were identified as independent risk factors for one-year mortality. (4) Conclusions: This study urges a critical examination of the justification for multivalve surgeries in high-risk elderly patients, emphasizing a paradigm shift. It advocates for interdisciplinary collaboration and innovative strategies, such as staged hybrid procedures, to improve therapeutic approaches for this challenging patient group to achieve a better therapeutic outcome for these patients.

## 1. Introduction

Life expectancy has increased in Western countries. The proportion of the population in Europe older than 80 has grown consistently and is forecasted to be 7.2% by 2030 [1]. Consequently, over the past decade, cardiac surgery has faced a steady increase in surgical intervention in geriatric patients. In numbers, the percentage of octogenarians in Germany requiring cardiac surgery rose from 13.8% in 2012 to 20.7% in 2021 [2]. Octogenarians occupy a very challenging role due to their unique age-related physiology, multiple comorbidities, and their exposure to complex chronic therapies. These result in a decompensated state of general health. Cardiovascular disease, in particular, is responsible for the deterioration of the general health of octogenarians. The incidence of cardiovascular disease (CVD) rises to 86% among those aged over 80 years [3]. Degenerative valvular heart disease is a non-negligible component of these cardiovascular diseases, which, in the elderly, often affects more than one heart valve, a condition also known as multivalvular disease (MVD). The term “multivalvular disease” describes the combination of stenotic or regurgitant lesions or both in more than one cardiac valve [4]. The Euro Heart Survey, after examining 5001 patients from 92 centers in 25 European countries, concluded that 25.4% of patients older than 70 years had MVD [5]. A study of 623,039 patients who underwent heart valve surgery between 1993 and 2007 found that 15.0% were octogenarians, and 10.9% had surgery for MVD [6]. However, the overall impact of cardiac surgery, including the extent of indicated surgery, the use of cardiopulmonary bypass (CPB), general anesthesia, and consecutive perioperative hemodynamic challenges during surgery and postoperative management in the intensive care unit, may detrimentally affect the outcome of octogenarian patients. In addition, recompensating therapy in the ICU places significant demands on ICU capacity, raising critical economic and ethical concerns. Against this backdrop, the most important question at the end of the day remains: is it worth the effort?

In the past, there have been publications looking at the outcome of heart surgery in octogenarians [7,8,9,10,11,12,13,14]. However, studies on the outcomes of cardiac surgery treatments for MVD in octogenarians are underrepresented and have so far only provided short-term results. The primary aim of this study was to fill this gap by retrospectively examining short-term and mid-term data from octogenarians with MVD who underwent cardiac surgery. The secondary aim was to delineate the predictors influencing in-hospital and one-year mortality within the same cohort of patients.

## 2. Patients and Methods

### 2.1. Inclusion and Exclusion Criteria

To define as homogeneous a cohort of patients as possible, the inclusion and exclusion criteria were strictly selected. The present study targeted octogenarians who had undergone multiple valve surgery (surgical intervention on at least two heart valves) via full sternotomy. Exclusion criteria were minimally invasive access routes (upper partial sternotomy, right anterolateral thoracotomy, and right transaxillary thoracotomy access), redo or emergency surgeries, preoperative cardiopulmonary resuscitation, concomitant coronary artery bypass grafting, and recent or active endocarditis.

### 2.2. Study Design and Ethical Statement

This study is a single-center, retrospective observational cohort analysis of consecutive octogenarian patients who underwent multiple valve surgery using the conventional full-sternotomy approach. Data were retrospectively extracted from the hospital database and consecutively deidentified, whereas the data acquisition was performed as part of routine patient care and treatment procedures. No specific written consent was obtained for this retrospective observational study since all patients’ data were anonymized and de-identified prior to analysis. The data in the follow-up period included outpatient clinical records from general practitioners or cardiologists and patient interviews. Data in the follow-up period were available for the entire study cohort. The primary postoperative outcomes, or endpoints, in this study were defined as major adverse cardio-cerebral events, encompassing perioperative myocardial infarction, perioperative stroke, and all-cause in-hospital mortality. In addition to the primary postoperative outcomes of major adverse cardio-cerebral events, secondary postoperative outcomes included a comprehensive assessment of other morbidities and complications arising after the surgical procedure. This research underwent evaluation and received approval from the Ethics Committee of the local institution (EK-Nr. 298092012 and EK-25006222016).

### 2.3. Patients

**Figure 1:**
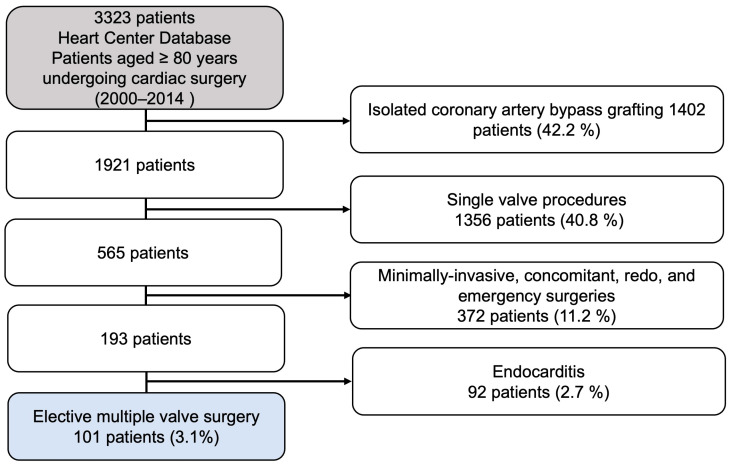
Flow diagram of the study population.

Since the year 2000, 3323 patients over the age of 80 have undergone cardiac surgery at our center. At our facility, indications for surgical procedures were made in accordance with the decision-making processes of the heart team, regardless of patient age. Surgical consent was obtained from all patients. After applying the inclusion and exclusion criteria, the final group of patients in the study consisted of 101 patients (Figure 1).

### 2.4. Statistical Analysis

All statistical analyses were performed using SAS JMP 12.2© (SAS Institute, Cary, NC, USA). Data are expressed as mean ± standard deviation (SD) in the case of continuous variables, whereas categorical variables are presented as absolute numbers and percentages. Besides patient baseline characteristics, outcome measures assessed were intraoperative parameters, postoperative disposition, and in-hospital mortality. The influence of relevant variables on in-hospital mortality and one-year mortality was investigated using a univariate logistic regression model. Variables demonstrating a tendency toward significance in univariate testing (*p* < 0.05) were included in a multivariate analysis. Mid-term survival analysis was performed using Kaplan–Meier’s curve.

### 2.5. Surgical Techniques

All surgical procedures were performed using a conventional median sternotomy. Extracorporeal circulation (ECC) was established via cannulation of the aorta ascendens and bicaval venous drainage of the superior and inferior vena cava or two-stage drainage by cannulation of the right atrium, depending on the surgeon’s preference. All surgical procedures were performed under normothermia. After cross-clamping the aorta, antegrade crystalloid cardioplegia was administered via the aorta ascendens. The left ventricular venting line was established via the right superior pulmonary vein. Atriotomies used were (I) left atrial through the interatrial groove, (II) trans-septal through the right atrium, or (III) extended superior trans-septal access as described by Guiraudon et al. [15]. The choice of the access route depended on the surgeon’s preference.

## 3. Results

### 3.1. Baseline Characteristics

According to the data provided in Table 1, the mean age of the cohort was 82.0 ± 1.9 years. Additionally, there was a higher prevalence of females in this investigated group, accounting for 66.3% (*n* = 67). The mean body mass index (BMI) was recorded as 25.7 ± 4.1 (kg/m^2^). The leading clinical symptom was dyspnea (*n* = 89, 88.1%). More than half of the patients (52.4%) in this observational study suffered from coronary artery disease (*n* = 53). Furthermore, a reduced left ventricular ejection fraction below 50% was observed in 39.6% (*n* = 40) of the patients. Preoperative manifest atrial fibrillation was already present in 53.6% (*n* = 51) of the individuals. The proportion of pulmonary arterial hypertension was similar, with a frequency of 53.5% (*n* = 54). The prevalence of diabetes mellitus within this cohort was 39.6% (*n* = 40). Notably, a significant portion of patients (*n* = 44) suffered from chronic kidney disease, indicating a prevalence rate of 43.6%. The mean logistic EuroSCORE recorded a value of 25.3 ± 19.5% with a corresponding EuroSCORE II of 16.8 ± 11.3% in the given study. The remaining data can be found in Table 1.

### 3.2. Surgical Procedures

**Figure 2:**
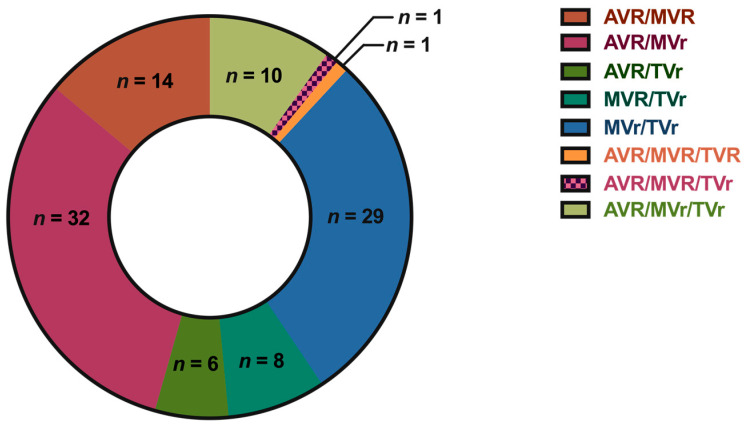
Distribution of patient cohort based on surgical interventions. Abbreviations: AVR, aortic valve replacement; MVR, mitral valve replacement; MVr, mitral valve repair; TVR, tricuspid valve replacement; TVr, tricuspid valve repair.

According to the findings illustrated in Figure 2, most patients (31.7%, *n* = 32) received aortic valve replacement along with concurrent mitral valve repair. The surgical intervention involving the mitral valve and concurrent reconstruction of the tricuspid valve was performed with the second highest frequency (28.7%, *n* = 29). The distribution of the other surgical procedures can be seen in Figure 2. The surgical procedure time was recorded as 165 ± 67 min. The mean cardiopulmonary bypass time observed in this study was 87 ± 21 min, with an accompanying mean aortic cross-clamp time of 66 ± 15 min.

### 3.3. In-Hospital Outcomes

**Figure 3:**
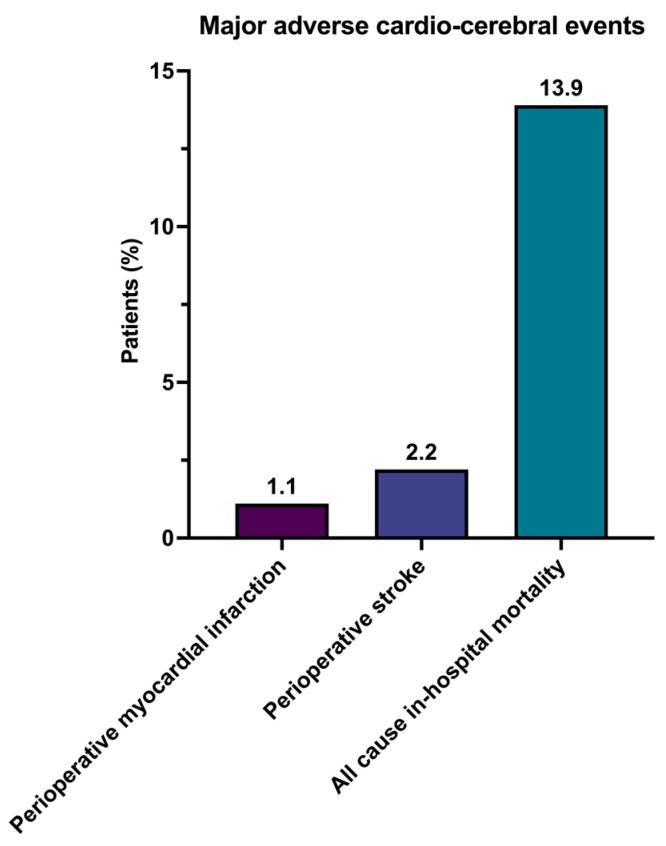
Primary postoperative outcomes in terms of major adverse cardio-cerebral events.

In the context of major adverse cardio–cerebral events, the incidence of perioperative myocardial infarction was 1.1%, while the stroke occurrence rate stood at 2.2%. An all-cause in-hospital mortality rate of 13.9% (*n* = 14) was observed (Figure 3).

**Figure 4:**
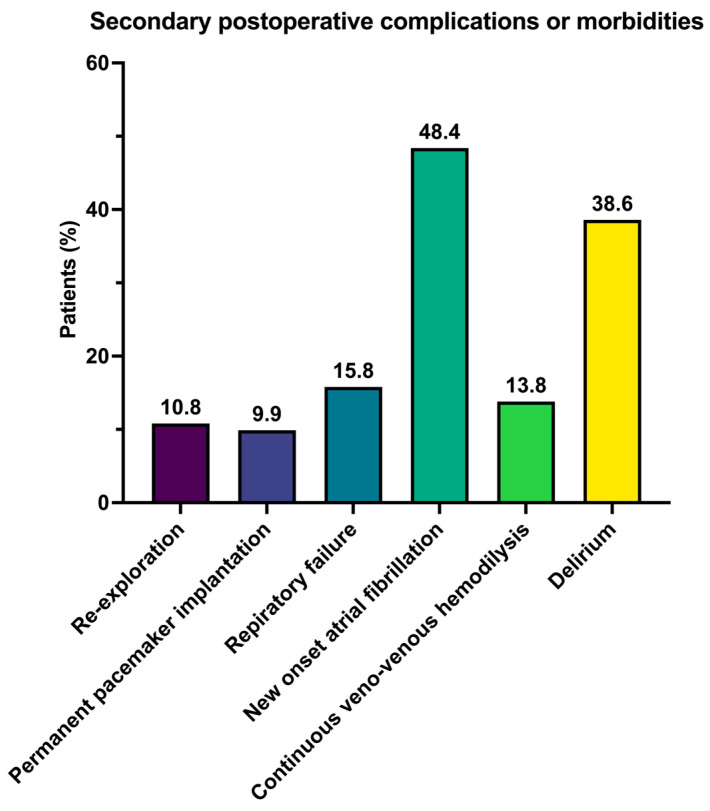
Secondary postoperative complications and morbidities.

Re-exploration due to bleeding or tamponade was necessary in 10.8% of cases. Postoperative conduction disturbances occurred in 9.9% of cases (*n* = 10), leading to the implantation of a permanent pacemaker system. Postoperative respiratory failure, defined as prolonged mechanical ventilation exceeding 72 h, re-intubation, and tracheotomy, occurred in 15.8% of patients (*n* = 16). New-onset atrial fibrillation occurred in 48.4% of the cases (*n* = 46). Acute renal failure, requiring continuous veno-venous hemodialysis, was present in 13.8% of the total cohort (*n* = 13). Postoperative delirium manifested in 38.6% of patients (*n* = 39). Among these cases, 10.8% (*n* = 10) specifically involved re-intubation. Notably, a prolonged duration of stay in the intensive care unit, exceeding 48 h, was observed in 62.4% of patients (*n* = 63). The mean length of hospitalization for the entire cohort was 16.0 ± 9.7 days (Figure 4).

**Figure 5:**
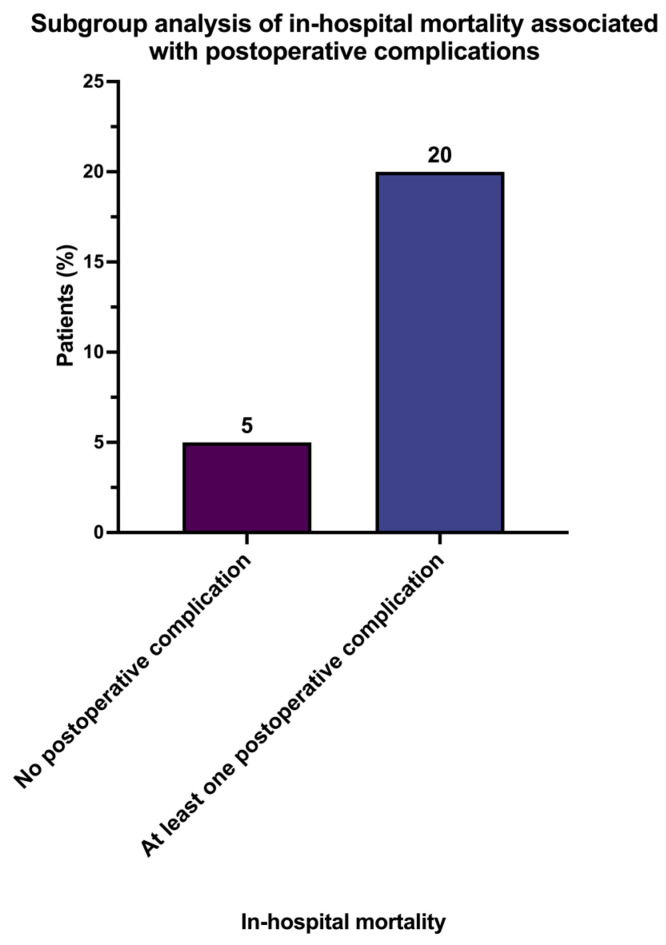
Subgroup analysis of in-hospital mortality associated with postoperative complications.

Subgroup analysis evaluating postoperative morbidity showed that a proportion of 39.6% (*n* = 40) of patients had a favorable outcome with no postoperative complications or morbidities. This group showed an in-hospital mortality rate of 5.0%. In the group of patients who experienced at least one or more postoperative complications, in-hospital mortality rose up to 20% (Figure 5).

### 3.4. Survival Analysis

**Figure 6:**
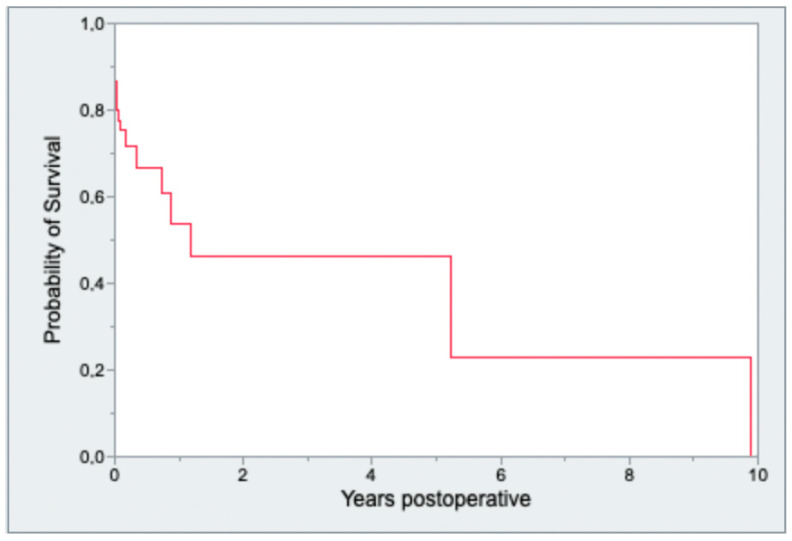
Kaplan–Meier survival analysis over an observational time period of ten years.

Analysis of follow-up data at one year showed that 29.7% (*n* = 30) died during the observation period; thus, the overall mortality was 43.8% (*n* = 44) after one year. As presented in Figure 6, the 6-month survival rate was 66.3%, and the one-year survival rate was 52.5%.

### 3.5. Univariate and Multivariate Analysis

In Table 2, the results of both univariate and multivariate analyses for in-hospital mortality, as well as one-year mortality, are presented.

#### 3.5.1. Univariate and Multivariate Analysis for In-Hospital Mortality

Univariate analysis revealed that among the demographic baseline characteristics, a lower BMI was associated with an increased risk of in-hospital mortality. Patients (*n* = 14, 13.9%) who experienced in-hospital mortality exhibited a mean BMI of 23.5 kg/m^2^, whereas those who survived the surgical procedure (*n* = 87, 86.1%) had a mean BMI of 26.0 kg/m^2^ (*p* = 0.04; odd ratio (OR), 1.2; confidence interval (CI), 1.1–2.9). In the univariate analysis, medical history data did not demonstrate any significant influence on in-hospital mortality. However, surgical procedure time had a notable univariate effect on in-hospital mortality, with patients experiencing in-hospital mortality having a mean surgical time of 252.1 min, compared to 151.7 min for those who survived (*p* < 0.01; OR, 2.1; CI, 1.7–3.6). Postoperative factors significantly associated univariately with in-hospital mortality were these five factors, including perioperative myocardial infarction (*p* = 0.04; OR, 1.8; CI, 1.1–3.1), perioperative stroke (*p* < 0.01; OR, 2.2; CI, 1.5–3.0), postoperative delirium (*p* = 0.04; OR, 1.4; CI, 1.1–2.5), continuous veno-venous dialysis (*p* < 0.01; OR, 3.8; CI 2.7–6.8), and re-exploration due to bleeding (*p* = 0.02, OR, 3.1; CI, 2.2–7.6). After adjusting for other relevant risk factors via multivariable analysis, surgical procedure time (*p* = 0.01; OR, 1.9; CI, 1.2–3.1), postoperative delirium (*p* < 0.01; OR, 2.4; CI, 1.3–2.7), and continuous veno-venous hemodialysis (*p* < 0.01; OR, 4.5; CI, 3.0–6.5) remained independent risk factors for reduced in-hospital survival.

#### 3.5.2. Univariate and Multivariate Analysis for One-Year Mortality

The univariate analysis concerning one-year mortality showed no significant impact on the demographic baseline characteristics. Pulmonary arterial hypertension was the sole parameter among the medical history variables that demonstrated a significant impact on one-year mortality (*p* = 0.02; OR, 1.3; CI, 1.2–3.1). The surgical procedure time showed a significant univariate impact on one-year mortality. Patients who died within one year had an average surgical time of 252.1 min, in contrast to 151.7 min for those who survived (*p* < 0.01; OR, 2.1; CI, 1.2–3.8). Postoperative clinical course parameters exhibiting a significant univariate association with in-hospital mortality included perioperative stroke (*p* = 0.04; OR, 1.6; CI, 1.1–2.4), postoperative delirium (*p* < 0.01; OR, 1.7; CI, 1.3–3.5), new-onset atrial fibrillation (*p* < 0.01; OR, 1.3; CI, 1.1–3.8), re-intubation (*p* = 0.04; OR, 1.8; CI, 1.1–4.7), continuous veno-venous dialysis (*p* = 0.04; OR, 2.5; CI, 1.5–5.6), pulmonary arterial hypertension (*p* = 0.03; OR, 1.4; CI, 1.1–2.9), and transfusion of packed red blood cells (*p* = 0.04; OR, 1.9; CI, 1.2–3.7). Following the adjustment for other pertinent risk factors in multivariable analysis, postoperative delirium (*p* < 0.001; OR, 1.6; CI, 1.2–1.8), postoperative new-onset atrial fibrillation (*p* < 0.001; OR, 1.4; CI, 1.4–2.3), re-intubation (*p* < 0.001; OR, 2.1; CI 1.7–2.4), and transfusion of any packed red blood cells (*p* < 0.001; OR, 1.3; CI, 1.1–1.6) remained independent risk factors associated with diminished one-year survival.

## 4. Discussion

The paucity of comprehensive diagnostic and therapeutic data for multivalve disease presents a formidable challenge for the treatment team, as evidence-based therapeutic interventions are hindered by the current lack of robust evidence [16,17,18]. However, evidence-based medicine encounters significant limitations when addressing octogenarians affected by multivalvular disease. This study aims to illuminate this obscure corner of medicine. In this investigation, we comprehensively studied a cohort of 101 consecutive octogenarian patients with multiple valvular heart diseases, leading to complex structural heart conditions and a high operative risk profile, who underwent surgical treatment. The main findings of this study can be summarized as follows:

- Mortality increased fourfold once at least one postoperative complication occurred.
- Octogenarians face a high in-hospital mortality rate of 13.9%.
- Nearly half (43.8%) of the octogenarian patients who underwent multiple valve surgery died after one year.
- In our investigation, postoperative delirium emerged as a significant independent risk factor, contributing not only to an elevated risk of in-hospital mortality but also to a higher likelihood of one-year mortality. Additionally, prolonged surgical procedure time was identified as an independent risk factor associated with increased in-hospital mortality. While continuous veno-venous hemodialysis showed an independent impact on in-hospital mortality, it did not exhibit a significant effect on one-year mortality. Furthermore, both re-intubation and the transfusion of packed red blood cells were identified as independent risk factors for one-year mortality.

Over the past decade, a continued rise in the number of elderly patients seeking cardiac surgical interventions has been observed [2]. Currently, cardiac procedures are conducted on 29.8% of patients falling within the age range of 70 to 79 years, while 20.7% of these procedures are performed on individuals in their eighties or nineties [2]. Regarding this progression, there are presently no indications suggesting an imminent reversal of this trend. Several research studies have assessed the impact of age on cardiac surgery outcomes and reported the feasibility of cardiac surgery in octogenarians [9,11,14,19,20,21,22]. As industrialized nations experience a continuous upward trend in demographic aging, the significance of this subject will gain even more prominence in the future. Despite the correlation between advancing age and mortality rates, the overall observed mortality in cardiac surgical procedures has remained consistently low, even with a marginal decline noted over the past decade [2,12,13]. Nevertheless, it is crucial to acknowledge the reality that patients aged 75 and above exhibit a significantly higher susceptibility to experiencing notable postoperative complications, including acute renal injury, respiratory failure, and perioperative stroke [23]. Furthermore, postoperative morbidity for older patients is contingent upon the specific type of cardiac surgical procedure being performed. Considering these aspects, it should be kept in mind that octogenarians who undergo combined procedures have a higher postoperative morbidity [10].

The association of surgical procedure time with mortality as an independent risk factor is anticipated, given its frequent connection with more intricate surgical procedures, resulting in prolonged cardiopulmonary bypass time (CPBT) and aortic cross-clamp time (ACCT). While the isolated association between surgical procedure time and increased mortality has not been specifically described in previous studies focusing on octogenarians undergoing cardiac surgical procedures, the age-independent correlation between mortality and longer CPBT and ACCT has been well documented in the literature [24,25,26].

In line with the findings of previous studies, postoperative delirium was identified as an independent factor for in-hospital mortality. However, the data from this study also demonstrated a sustained impact of postoperative delirium as an independent risk factor for one-year mortality.

Preliminary studies have yielded inconsistent findings concerning the association between new-onset atrial fibrillation and long-term mortality following cardiac surgery [27,28,29,30]. While many studies have focused on patients undergoing coronary bypass surgery, limited data exist on the association between new-onset atrial fibrillation and mid- or long-term mortality following valve or multivalve surgery. Our study indicates that new-onset atrial fibrillation has no impact on in-hospital mortality but does influence one-year mortality.

Consistent with insights gleaned from prior studies, the findings of this study underscore the susceptibility of this demanding and complex patient cohort, thereby prompting a legitimate inquiry into the justification of surgical intervention for octogenarians with multiple valve disease. Thus, it is imperative to raise the question of whether the feasibility of performing cardiac surgery on this vulnerable group of patients, characterized by intricate valvular and consequential structural heart conditions, should be re-evaluated. Despite the recognized comorbidities that pose significant risks, several factors need to be considered when assessing a patient’s inoperability. In an alternative formulation, declaring this patient cohort inoperable fails to adequately acknowledge the wide array of therapeutic options available today and the consequent imperative to provide patients with appropriate treatment strategies rather than leaving them to endure their suffering in isolation. However, the prerequisite for this is to think “out of the box.” This proposition calls for progressive-minded physicians who are willing to challenge the established boundaries between disciplines. This raises the question: What could be a viable approach to a solution? The first step in addressing this question in a forward-thinking way is to recognize that the traditional approach of a single procedure conducted by a single medical discipline may no longer fully meet the needs of this complex, diseased patient cohort. Thus, more than ever, there is a heightened demand for interdisciplinary teams to implement holistic approaches, wherein a tailored and nuanced multidisciplinary treatment strategy can be devised. The interdisciplinary team’s expertise in employing a comprehensive range of minimally invasive techniques, catheter-based interventions, and conventional surgical procedures is poised to facilitate the development of tailored treatment plans for these vulnerable high-risk patients. More specifically, hybrid therapy approaches, yet to be fully explored, represent an underexplored interdisciplinary strategy in treating high-risk patients to transform a single high-risk open cardiac surgical procedure into two less risky procedures. This may imply that the leading valve defect can be repaired using a minimally invasive surgical therapy approach, followed by addressing additional valve defects in a subsequent step via catheter-based interventions. The efficacy and untapped potential of staged hybrid procedures for high-risk patient groups, such as octogenarians with multivalve disease, have not been sufficiently researched. Initiating this research necessitates a fundamental re-evaluation, even a paradigm shift in therapeutic strategies, to enable staged hybrid procedures via interdisciplinary collaboration.

To effectively and concretely address the challenges posed by multivalve disease in octogenarians, a multidisciplinary, staged, hybrid therapeutic approach is a potential solution. Considering the three pillars mentioned, in the first step, there should be a concerted effort to establish close multidisciplinary collaboration, primarily involving geriatricians, cardiologists, and cardiac surgeons. Since current guidelines may not be adequate for managing the varying clinical scenarios of multivalve disease in octogenarians, the expertise of a multidisciplinary team is of paramount importance [31]. This collaboration aims to regularly assess the patient’s disease progression. Possibly, this way, deteriorations can be detected earlier, and the therapeutic direction can be adjusted accordingly. Once disease progression has manifested, the patient must be re-evaluated in a multidisciplinary manner, considering all individual risk factors, to determine whether a catheter-based or surgical therapeutic approach is warranted. If surgical treatment is chosen as the therapy of choice for any reason, the goal should be to initially address the leading valve disease via minimally invasive techniques whenever possible, aiming to keep the operative risk manageable. Subsequently, the patient can be initially medically managed in a multidisciplinary manner and re-evaluated at regular intervals. If necessary, the other affected heart valves may be addressed in a further hybrid therapy approach with a catheter-based intervention. In this context, it is crucial to emphasize the pivotal role of pharmacological therapy for heart failure, particularly in collaboration between geriatric and cardiological disciplines.

## 5. Limitations

First and foremost, this study is constrained by its single-center retrospective design, limiting the generalizability of findings to a broader population. Second, the absence of a control group in the investigation of a specific cohort of octogenarian patients with multiple valvular heart diseases and a high surgical risk profile is a noteworthy limitation. The retrospective nature of the study introduces inherent constraints related to data availability, completeness, and potential biases. Variations in surgical techniques and advancements over the study period may also impact the study’s results. Furthermore, the focus on short- and one-year outcomes restricts a comprehensive understanding of the long-term effects of surgical interventions on this challenging patient group, highlighting the need for extended follow-up investigations to address this limitation.

## 6. Conclusions

In summary, the increasing number of elderly patients opting for cardiac surgeries, especially those aged 80 and above, necessitates a reassessment of the feasibility and justification of these procedures. While overall mortality rates remain low, the heightened susceptibility of patients aged 80 and above to postoperative complications raises concerns. This study on octogenarians with multiple valvular heart diseases and high operative risk reveals a fourfold increase in mortality with postoperative complications and a significant one-year mortality rate. These findings warrant a critical examination of the justification for surgical interventions in this challenging patient cohort.

Given these concerns, a forward-thinking approach should involve interdisciplinary teams and explore promising yet underexplored strategies, such as staged hybrid procedures combining minimally invasive surgery and catheter-based intervention. This paradigm shift requires collaboration across disciplines to improve therapeutic strategies for high-risk elderly patients. The insights from this study suggest that the time for a paradigm shift in cardiac care for older patients is indeed justified.

## Figures and Tables

**Table 1:** Patient baseline characteristics.

Variables	
Baseline demographic characteristics	
Age (years), mean ± SD	82.0 ± 2.0
Sex (female), *n* (%)	67 (66.3)
Body mass index (kg/m^2^), mean ± SD	25.7 ± 4.1
Clinical symptoms and signs	
NYHA class III or IV, *n* (%)	78 (77.2)
Dyspnea	89 (88.1)
Anginal complaints	11 (10.9)
Medical history	
Coronary artery disease, *n* (%)	53 (52.4)
Preoperative LVEF < 50%, *n* (%)	40 (39.6)
Preoperative atrial fibrillation, *n* (%)	51 (53.6)
Chronic obstructive pulmonary disease, *n* (%)	7 (6.9)
Pulmonary arterial hypertension, *n* (%)	54 (53.5)
Chronic kidney disease, *n* (%)	44 (43.6)
Extracardiac arteriopathy, *n* (%)	14 (13.9)
Diabetes mellitus, *n* (%)	40 (39.6)
Valve Pathology	
Aortic valve pathology, *n* (%)	73 (72.2)
Aortic valve stenosis, *n* (%)	58 (79.5)
Aortic valve regurgitation, *n* (%)	15 (20.5)
Mitral valve pathology, *n* (%)	95 (94.1)
Mitral valve stenosis, *n* (%)	6 (6.3)
Mitral valve regurgitation, *n* (%)	89 (93.7)
Tricuspid valve pathology, *n* (%)	74 (73.2)
Tricuspid valve stenosis, *n* (%)	1 (1.3)
Tricuspid valve regurgitation, *n* (%)	73 (98.7)
Surgical risk stratification	
Logistic EuroSCORE (%), mean ± SD	25.3 ± 19.5
EuroSCORE II (%), mean ± SD	16.8 ± 11.3

**Table 2:** Univariate and multivariate analysis for in-hospital and one-year mortality.

Variables	In-Hospital Mortality				One-Year Mortality			
	Univariable Analysis		Multivariable Analysis		Univariable Analysis		Multivariable Analysis	
	OR (95% CI)	*p*	OR (95% CI)	*p*	OR (95% CI)	*p*	OR (95% CI)	*p*
Baseline demographic characteristics								
Age (years)	-	0.110	-	0.210	-	0.400	-	0.170
Sex (female)	-	0.270	-	0.140	-	0.100	-	0.460
Body mass index (kg/m^2^)	1.2 (1.1, 2.9)	0.040 *	-	0.170	-	0.990	-	0.950
Medical history								
Coronary artery disease	-	0.280	-	0.360	-	0.410	-	0.520
Preoperative LVEF < 50%	-	0.090	-	0.110	-	0.110	-	0.410
Preoperative atrial fibrillation	-	0.170	-	0.170	-	0.630	-	0.570
Chronic obstructive pulmonary disease	-	0.230	-	0.450	-	0.330	-	0.480
Pulmonary arterial hypertension	-	0.090	-	0.249	1.3 (1.2, 3.1)	0.020 *	-	0.180
Chronic kidney disease	-	0.560	-	0.910	-	0.900	-	0.920
Extracardiac arteriopathy	-	0.400	-	0.110	-	0.690	-	0.520
Diabetes mellitus	-	0.950	-	0.210	-	0.360	-	0.150
Procedural parameters								
Surgical procedure time (min)	2.1 (1.7, 3.6)	<0.010 *	1.9 (1.2, 3.1)	0.010 *	2.1 (1.2, 3.8)	<0.001 *	-	0.680
Parameters of postoperative clinical course								
Perioperative myocardial infarction	1.8 (1.1, 3.1)	0.040 *	-	0.999	-	0.190	-	0.490
Perioperative stroke	2.2 (1.5, 3.0)	<0.010 *	-	0.991	1.6 (1.1, 2.4)	0.040 *	-	0.999
Postoperative delirium	1.4 (1.1, 2.5)	0.040 *	2.4 (1.3, 2.7)	<0.01 *	1.7 (1.3, 3.5)	<0.010 *	1.6 (1.2, 1.8)	<0.001 *
New-onset atrial fibrillation	-	0.170	-	0.174	1.3 (1.1, 3.8)	<0.010 *	1.8 (1.4, 2.3)	<0.001 *
Re-intubation	-	0.340	-	0.549	1.8 (1.1, 4.7)	0.040 *	2.1 (1.7–2.4)	<0.001 *
Continuous veno-venous hemodialysis	3.8 (2.7, 6.8)	<0.010 *	4.5 (3.0, 6.5)	<0.010 *	2.5 (1.5, 5.6)	0.040 *	-	0.950
Pulmonary arterial hypertension	-	0.090	-	0.249	1.4 (1.1, 2.9)	0.030 *		0.100
Re-exploration	3.1 (2.2, 7.6)	0.020 *	-	0.500	-	0.123	-	0.330
Transfusion of PRBC (any)	-	0.080	-	0.131	1.9 (1.2, 3.7)	0.040 *	1.3 (1.1, 1.6)	<0.001 *

Note: Bold and italic values indicate statistical significance: *, *p* ≤ 0.05. Abbreviations: OR, odd ratio; CI, confidence interval; PRBC, packed red blood cells; LVEF, left ventricular ejection fraction.

## Data Availability

The data presented in this study are available upon request from the corresponding author. The data are not publicly available due to ethical regulations.
